# Supplementary material for: Interventions Designed to Support Physical Activity and Disease Prevention for Working from Home: A Scoping Review
Source: Int J Environ Res Public Health. 2022 Dec 21;20(1):73. doi: 10.3390/ijerph20010073 (PMC9819910; doi:10.3390/ijerph20010073)
Supplement: Supplementary file 1 [file ijerph-20-00073-s001.zip › ijerph-2027940-supplementary.pdf]

## Supplementary File S1 – scoping review search strategy

This comprehensive strategy followed other reviews of emerging literature (1, 2).

### Published and grey literature

| Database | Search term |
|---|---|
| **Scopus** | TITLE-ABS-KEY ( telecommut* OR "work* from home" OR "work* at home" OR telework* OR ( workplace* W/1 flexib* ) OR "flexible work* arrangement*" OR ( work* W/1 remote* ) OR "home based work*" OR "home working" OR "homeworking" OR "virtual office" OR ( work* W/1 ( virtual* OR digital* OR online OR distant*) ) OR "stay* home" OR "stay* at home" OR ( home W/1 ( confine* OR isolat* ) ) OR ( covid W/4 ( restrict* OR isolat* OR confine* ) ) ) AND TITLE-ABS-KEY ( "physical* activ*" OR "active travel*" OR exercise OR "sedentary behavio#r" OR sitting OR fitness OR sport* OR walk* OR cycling OR bicycl* OR "energy expenditure" OR "aerobic train*" OR "physical exertion" OR "resistance train*" OR "strength train*" ) AND TITLE-ABS-KEY ( intervention* OR initiative* OR experiment* OR evaluat* OR program* OR promot* OR policy OR strategy OR "prospective stud*" ) AND TITLE-ABS-KEY ( health* ) AND ( PUBYEAR > 2009 ) AND ( LIMIT-TO ( SRCTYPE,"j" ) ) AND ( LIMIT-TO ( LANGUAGE,"English" ) ) |
| **Web of Science** (core collection) | TS=(telecommut* OR “work* from home” OR “work* at home” OR telework* OR (workplace* NEAR/1 flexib*) OR “flexible work* arrangement*” OR “home working” OR "homeworking” OR “virtual office” OR (work* NEAR/1 (remote* OR virtual* OR digital* OR online OR distant*)) OR “stay* home“ OR "stay* at home" OR (home NEAR/1 (confine* OR isolat*)) OR (covid NEAR/4 (restrict* OR isolat* OR confine*))) AND TS=(intervention* OR initiative* OR experiment* OR evaluat* OR program* OR promot* OR policy OR strategy OR "prospective stud*") AND TS=(“physical* activ*” OR “active travel” OR exercise OR “sedentary behaviour” OR sitting OR fitness OR sport* OR walk* OR cycling OR bicycl* OR “energy expenditure” OR “physical exertion” OR “aerobic train*” OR “resistance train*” OR “strength train*”) AND TS=(health*)<br>*Filtered to:* Language = English; Doctype = Articles, review articles, early access, book chapters (for published literature sources) or Proceedings, editorial, data papers, letters, meting abstracts (grey literature sources); Timespan = 2010-01-01 – 2022-04-04 |
| **EBSCO host** (Rehabilitation & Sports Medicine Source, MEDLINE Complete, Psychology and Behavioral Sciences Collection, Applied Science & Technology Source | AB ( telecommut* OR "work* from home" OR "work* at home" OR telework* OR ( workplace* N1 flexib* ) OR "flexible work* arrangement*" OR "home working" OR "homeworking" OR "virtual office" OR ( work* N1 ( remote* OR virtual* OR digital* OR online OR distant* ) OR "stay* at home" OR ( covid N4 (restrict* OR isolat* OR confine* ) ) ) AND AB ( physical* activ* OR "active travel*" OR exercise OR "sedentary behavio?r" OR sitting OR fitness OR sport* OR walk* OR cycling OR bicycl* OR "energy expenditure" OR "aerobic train*" OR "physical exertion" OR "resistance train*" OR "strength train*" ) AND AB ( intervention* OR initiative* OR |

| | |
|---|---|
| Ultimate, Business Source Ultimate, CINAHL Complete, SPORTDiscus) | experiment* OR evaluat* OR program* OR promot* OR policy OR strategy OR "prospective stud*" ) AND AB ( health* )<br>*Filtered to:* Language = English; Pub date: 2010-01-01 – 2022-04-04;<br>For published literature search Source type: academic journals<br>For grey literature search Source type: magazines, trade publications, reports, conference materials |

| Source / database | Search term |
|---|---|
| **Mednar** | ( "work from home" OR telecommuting OR "flexible work" OR "online work" OR "virtual work") AND ( "physical activity" OR exercise OR cycling OR walking OR sitting ) AND ( policy OR initiative OR intervention OR program OR strategy ) AND health<br>*Filtered to*: Date = 2020-01-01 – 2022-04-04 |
| **Google** | ( "work from home" OR telecommuting OR "flexible work" OR "online work" OR "virtual work") AND ( "physical activity" OR exercise OR cycling OR walking OR sitting ) AND ( policy OR initiative OR intervention OR program OR strategy ) AND health<br>*Filtered to*: Date = 2020-01-01 – 2022-04-04 |
| **Targeted organisation websites** | *Search term:* "work from home" AND "physical activity"*<br>*Filtered to:* Date = 2020-2022 |
| | World Health Organization, https://apps.who.int/iris/<br>Centre For Disease Prevention and Control, US, https://www.cdc.gov<br>Department of Health, Australia, https://www.health.gov.au<br>Safework, Australia, https://www.safeworkaustralia.gov.au<br>Health and Safety Executive, UK, https://www.hse.gov.uk<br>Deloitte, https://www2.deloitte.com/kg/en.html<br>Forbes, https://www.forbes.com<br>Price Waterhouse Cooper, https://www.pwc.com<br>Gartner, https://www.gartner.com/en |

**References**

1. Abdi S, de Witte L, Hawley M. Emerging technologies with potential care and support applications for older people: review of gray literature. JMIR aging. 2020;3(2):e17286.

2. Godin K, Stapleton J, Kirkpatrick SI, Hanning RM, Leatherdale ST. Applying systematic review search methods to the grey literature: a case study examining guidelines for school-based breakfast programs in Canada. Systematic reviews. 2015;4(1):1-10.
